# [1,2-Bis(diisopropyl­phosphan­yl)ethane-κ^2^ *P*,*P*′](carbonato-κ^2^ *O*,*O*′)nickel(II)

**DOI:** 10.1107/S1600536813006521

**Published:** 2013-03-13

**Authors:** Illan Morales-Becerril, Marcos Flores-Alamo, Juventino J. Garcia

**Affiliations:** a Facultad de Química, Universidad Nacional Autónoma de México, México DF, 04510, Mexico

## Abstract

In the crystal of the title compound, [Ni(CO_3_)(C_14_H_32_P_2_)], the metal center in each of three independent mol­ecules shows slight tetra­hedral distortion from ideal square-planar coordination geometry, with angles between the normals to the planes defined by the *cis*-P—Ni—P and *cis*-O—Ni—O fragments of 3.92 (17), 0.70 (16) and 2.17 (14)° in the three mol­ecules. In the crystal, there are inter­molecular C—H⋯O hydrogen bonds that show a laminar growth in the *ab* plane.

## Related literature

For the synthesis and related structures, see: González-Sebastián *et al.* (2012 ▶); Cañavera-Buelvas *et al.* (2011 ▶); Castellanos-Blanco *et al.* (2011 ▶); Angulo *et al.* (2003 ▶); Dahlenburg & Kurth (2001 ▶). For applications of nickel complexes to catalytic systems, see: Vicic & Jones (1997 ▶); Arévalo & García (2010 ▶). For nickel compounds in CO_2_ activation, see: Anderson *et al.* (2010 ▶); Aresta *et al.* (1975 ▶).
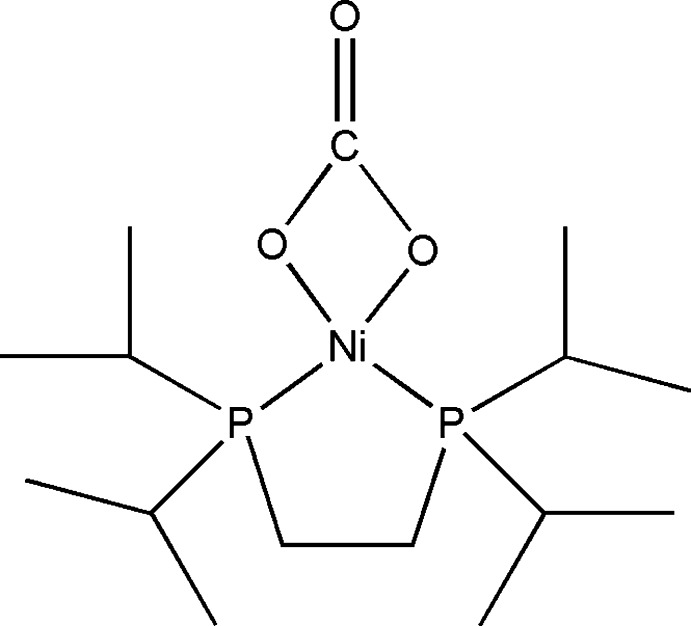


## Experimental

### 

#### Crystal data

[Ni(CO_3_)(C_14_H_32_P_2_)]
*M* *_r_* = 381.06
Monoclinic, 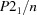

*a* = 8.4974 (4) Å
*b* = 46.582 (2) Å
*c* = 14.7342 (7) Å
β = 103.618 (4)°
*V* = 5668.2 (5) Å^3^
*Z* = 12
Mo *K*α radiation
μ = 1.20 mm^−1^
*T* = 130 K
0.33 × 0.06 × 0.03 mm

#### Data collection

Oxford Diffraction Xcalibur (Atlas, Gemini) diffractometer
Absorption correction: analytical (*CrysAlis PRO*; Oxford Diffraction, 2010 ▶) *T*_min_ = 0.813, *T*_max_ = 0.965
42978 measured reflections
10329 independent reflections
7642 reflections with *I* > 2σ(*I*)
*R*_int_ = 0.088

#### Refinement

*R*[*F* ^2^ > 2σ(*F* ^2^)] = 0.062
*wR*(*F* ^2^) = 0.096
*S* = 1.09
10329 reflections
592 parameters
H-atom parameters constrained
Δρ_max_ = 0.57 e Å^−3^
Δρ_min_ = −0.59 e Å^−3^

### 

Data collection: *CrysAlis CCD* (Oxford Diffraction, 2009 ▶); cell refinement: *CrysAlis RED* (Oxford Diffraction, 2009 ▶); data reduction: *CrysAlis RED*; program(s) used to solve structure: *SHELXS97* (Sheldrick, 2008 ▶); program(s) used to refine structure: *SHELXL97* (Sheldrick, 2008 ▶); molecular graphics: *ORTEP-3 for Windows* (Farrugia, 2012 ▶); software used to prepare material for publication: *WinGX* (Farrugia, 2012 ▶).

## Supplementary Material

Crystal structure: contains datablock(s) global, I. DOI: 10.1107/S1600536813006521/ru2049sup1.cif

Structure factors: contains datablock(s) I. DOI: 10.1107/S1600536813006521/ru2049Isup2.hkl

Additional supplementary materials:  crystallographic information; 3D view; checkCIF report

## Figures and Tables

**Table 1:** Selected bond lengths (Å)

Ni1*A*—O1*A*	1.879 (2)
Ni1*A*—O2*A*	1.885 (3)
Ni1*A*—P1*A*	2.1390 (12)
Ni1*A*—P2*A*	2.1460 (11)
Ni1*B*—O2*B*	1.887 (2)
Ni1*B*—O1*B*	1.890 (3)
Ni1*B*—P2*B*	2.1399 (12)
Ni1*B*—P1*B*	2.1415 (11)
Ni1*C*—O2*C*	1.877 (3)
Ni1*C*—O1*C*	1.889 (2)
Ni1*C*—P2*C*	2.1433 (10)
Ni1*C*—P1*C*	2.1481 (12)

**Table 2:** Hydrogen-bond geometry (Å, °)

*D*—H⋯*A*	*D*—H	H⋯*A*	*D*⋯*A*	*D*—H⋯*A*
C5*A*—H5*A*1⋯O3*A* ^i^	0.98	2.70	3.670 (5)	169
C4*A*—H4*A*3⋯O1*A* ^i^	0.98	2.69	3.448 (5)	134
C8*C*—H8*C*1⋯O3*C* ^ii^	0.98	2.71	3.595 (5)	150
C10*B*—H10*F*⋯O3*B* ^ii^	0.98	2.52	3.335 (5)	141
C1*A*—H1*A*2⋯O3*B* ^ii^	0.99	2.23	3.204 (5)	168
C1*C*—H1*C*2⋯O3*A* ^iii^	0.99	2.50	3.443 (5)	159
C9*C*—H9*C*⋯O2*A* ^iii^	1.00	2.48	3.455 (5)	165
C1*B*—H1*B*1⋯O3*C* ^iv^	0.99	2.50	3.452 (5)	161
C6*B*—H6*B*⋯O3*C* ^iv^	1.00	2.60	3.516 (5)	153

Symmetry codes: (i) 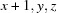; (ii) 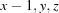; (iii) 
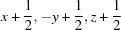
; (iv) 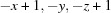.

**Fig. 1.**
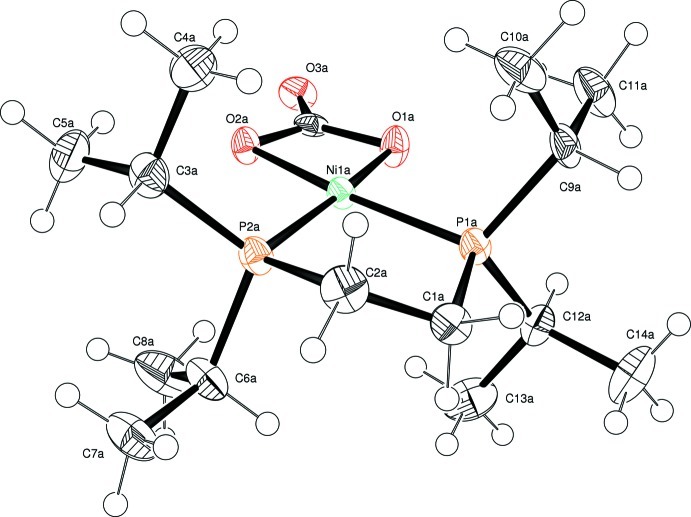
The molecular structure of the title compound. Displacement ellipsoids are drawn at the 50% probability level and H atoms are shown as circles of arbitrary size.

**Fig. 2.**
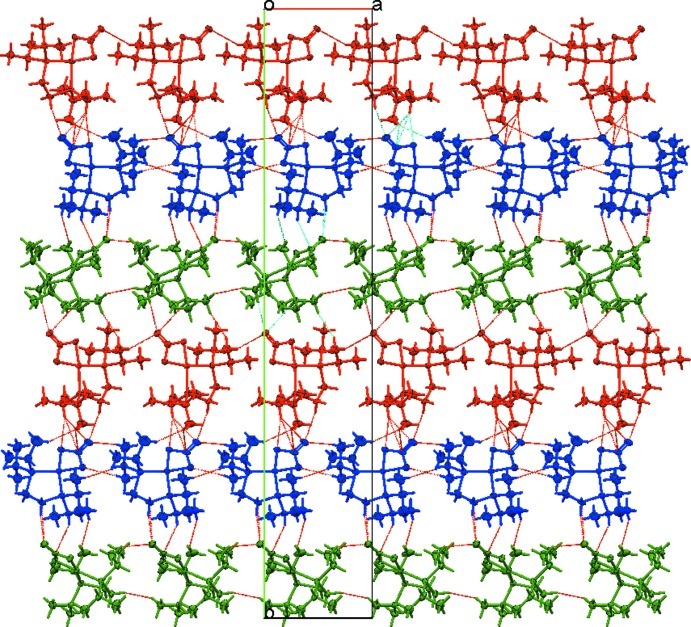
Crystal structure of the title compound viewed along the c axis, showing the short contacts between the symmetry equivalent for molecule A (blue), molecule B (green) and molecule C (red) extending along the a-b plane.

## supplementary crystallographic information

### Comment

Nickel compounds are highly active in CO_2_ activation (Aresta *et al.*, 1975, Anderson *et al.*, 2010), to produce carbonyl and carbonato derivatives. We recently published the complex [(*dippe*)Ni(CO_3_)] with a methanol solvate (González-Sebastián *et al.*, 2012).

The asymmetric unit consists of three [(*dippe*)Ni(CO_3_)] discrete molecules of the neutral complex (Figure 1). The Ni(II) atom is coordinated by two P atoms of *dippe* ligand and two oxygen atoms of the carbonato anion. The metal center in 3 independent molecules *A, B* and *C* of [(*dippe*)Ni(CO_3_)] shows slight tetrahedral distortion from ideal square planar coordination geometry, with the angle between the normals to the planes defined by the two *cis*-P–Ni–P and *cis*- O–Ni–O fragments of 3.92 (17), 0.70 (16) and 2.17 (14)° respectively, these being larger than the limiting value of 0° for square-planar coordination in [(*dippe*)Ni(CO)_2_]CH_3_OH (González-Sebastián *et al.* 2012). Additionally the Ni(II) atom is situated 0.040 (1), 0.0057 (9), 0.0095 (9) Å above the P1/P2/O1/O2 plane in *A, B* and *C* molecules respectively. These deviations from planarity, which can be attributed to some steric efect of the *dippe* ligand and intermolecular interactions of the carbonato ligand, are somewhat shorter than the distortion from ideal square-planar coordination geometry observed on [(*dippe*)Ni(carbazole)_2_ (Cañavera-Buelvas *et al.*, 2011) and [(*dippe*)NiCl_2_] (Castellanos-Blanco *et al.*, 2011,) complexes where the NiCl2/NiP2 dihedral angles of 15.32 and 10.01 ° respectively, and similar to the distortion from ideal square-planar coordination geometry observed for related [(dcpe)NiCl_2_] (Angulo *et al.*, 2003) and [(1*S*,2*S*)- C_5_H_8_{P(C_6_H_1_1)_2_}_2_NiCl_2_] (Dahlenburg & Kurth, 2001) complexes, where the NiCl2/NiP2 dihedral angles of 3.96 and 5.37°, respectively.

In the crystal packing, there are intermolecular contacts of the type hydrogen bond (Table 2) mainly between the carbon donor atom of the dippe to O oxygen atom acceptor of the metallic complex mainly. The C5A-H5A1···O3A (2.7 Å) and C4A-H4A3···O1A (2.69 Å) intermolecular interactions in molecule *A* forming a motif graph *R_2_^2^(8)* along the *a* axes, while the C10B—H10F···O3B (2.52 Å) and C8C-H8C1···O3C (2.71 Å) intermolecular interactions in molecules *B* and *C* forming a *C(8)* motif along to *c* axis. All these interactions show a laminar growing in the *a*, *b* plane (Figure 2).

### Experimental

The compound [(*dippe*)NiCl_2_] (98.0 mg, 0.25 mmol) was slowly added into a solution of commercially available KOH (28.0 mg, 0. 50 mmol) in H_2_O (5 ml) under constant stirring at room temperature. After 15 min of reaction, a red solution was observed. At this point the reaction mixture was evaporated to dryness under vacuum and the obtained red-wine residue was re-dissolved in THF (5 mL) and filtrated *via* cannula using a Schlenk flask. After a couple of days of cooling in the dry-box fridge at -30 °C, yellow crystals suitable for *X* ray diffraction studies were obtained.

The yellow crystals for complex [(*dippe*)Ni(CO_3_)] displayed a singlet in ^31^P{^1^H} NMR (THF-d_8_): 87.8 p.p.m., clearly this product raised from the carbonate present in the commercial KOH.

### Refinement

H atoms attached to C atoms were placed in geometrically idealized positions, and refined as riding on their parent atoms, with C—H distances fixed to 0.98 (methyl CH_3_), 0.99 (methylene CH_2_) and 1.00 Å (methine CH), and with *U*_iso_(H) = 1.5Ueq(methyl C) or 1.2Ueq(C).

### Crystal data

**Table:**

[Ni(CO_3_)(C_14_H_32_P_2_)]	*F*(000) = 2448
*M**_r_* = 381.06	*D*_x_ = 1.34 Mg m^−^^3^
Monoclinic, *P*2_1_/*n*	Mo *K*α radiation, λ = 0.71073 Å
*a* = 8.4974 (4) Å	Cell parameters from 6343 reflections
*b* = 46.582 (2) Å	θ = 3.4–29.5°
*c* = 14.7342 (7) Å	µ = 1.20 mm^−^^1^
β = 103.618 (4)°	*T* = 130 K
*V* = 5668.2 (5) Å^3^	Needle, pale yellow
*Z* = 12	0.33 × 0.06 × 0.03 mm

### Data collection

**Table:**

Oxford Diffraction Xcalibur (Atlas, Gemini) diffractometer	10329 independent reflections
Graphite monochromator	7642 reflections with *I* > 2σ(*I*)
Detector resolution: 10.4685 pixels mm^-1^	*R*_int_ = 0.088
ω scans	θ_max_ = 25.4°, θ_min_ = 3.4°
Absorption correction: analytical (*CrysAlis PRO*; Oxford Diffraction, 2010)	*h* = −10→10
*T*_min_ = 0.813, *T*_max_ = 0.965	*k* = −56→55
42978 measured reflections	*l* = −13→17

### Refinement

**Table:**

Refinement on *F*^2^	Primary atom site location: structure-invariant direct methods
Least-squares matrix: full	Secondary atom site location: difference Fourier map
*R*[*F*^2^ > 2σ(*F*^2^)] = 0.062	Hydrogen site location: inferred from neighbouring sites
*wR*(*F*^2^) = 0.096	H-atom parameters constrained
*S* = 1.09	*w* = 1/[σ^2^(*F*_o_^2^) + (0.0167*P*)^2^ + 0.6693*P*] where *P* = (*F*_o_^2^ + 2*F*_c_^2^)/3
10329 reflections	(Δ/σ)_max_ = 0.001
592 parameters	Δρ_max_ = 0.57 e Å^−^^3^
0 restraints	Δρ_min_ = −0.59 e Å^−^^3^

### Special details

**Table:**

Geometry. All s.u.'s (except the s.u. in the dihedral angle between two l.s. planes) are estimated using the full covariance matrix. The cell s.u.'s are taken into account individually in the estimation of s.u.'s in distances, angles and torsion angles; correlations between s.u.'s in cell parameters are only used when they are defined by crystal symmetry. An approximate (isotropic) treatment of cell s.u.'s is used for estimating s.u.'s involving l.s. planes.
Refinement. Refinement of *F*^2^ against ALL reflections. The weighted *R*-factor *wR* and goodness of fit *S* are based on *F*^2^, conventional *R*-factors *R* are based on *F*, with *F* set to zero for negative *F*^2^. The threshold expression of *F*^2^ > 2σ(*F*^2^) is used only for calculating *R*-factors(gt) *etc*. and is not relevant to the choice of reflections for refinement. *R*-factors based on *F*^2^ are statistically about twice as large as those based on *F*, and *R*- factors based on ALL data will be even larger.

### Fractional atomic coordinates and isotropic or equivalent isotropic displacement parameters (Å^2^)

**Table:**

	*x*	*y*	*z*	*U*_iso_*/*U*_eq_	
C1A	0.0622 (4)	0.19012 (9)	0.3969 (3)	0.0260 (11)	
H1A1	0.0615	0.1964	0.4611	0.031*	
H1A2	0.0722	0.169	0.3969	0.031*	
C2A	0.2069 (5)	0.20374 (10)	0.3671 (3)	0.0317 (12)	
H2A1	0.2366	0.1919	0.3179	0.038*	
H2A2	0.3014	0.2046	0.4212	0.038*	
C3A	0.2999 (5)	0.24984 (10)	0.2547 (3)	0.0295 (11)	
H3A	0.4108	0.2472	0.2957	0.035*	
C4A	0.2828 (5)	0.22929 (11)	0.1706 (3)	0.0365 (12)	
H4A1	0.176	0.2318	0.1283	0.055*	
H4A2	0.2944	0.2094	0.1929	0.055*	
H4A3	0.3672	0.2336	0.1373	0.055*	
C5A	0.2843 (5)	0.28071 (11)	0.2228 (4)	0.0429 (14)	
H5A1	0.3655	0.2849	0.1872	0.064*	
H5A2	0.3015	0.2934	0.2774	0.064*	
H5A3	0.1757	0.2839	0.1833	0.064*	
C6A	0.1872 (5)	0.26167 (10)	0.4291 (3)	0.0334 (12)	
H6A	0.1319	0.2511	0.4719	0.04*	
C7A	0.3645 (5)	0.26406 (12)	0.4821 (3)	0.0446 (14)	
H7A1	0.4255	0.2745	0.444	0.067*	
H7A2	0.4104	0.2448	0.4955	0.067*	
H7A3	0.3714	0.2744	0.5409	0.067*	
C8A	0.1082 (6)	0.29068 (12)	0.4154 (4)	0.0540 (16)	
H8A1	0.0955	0.298	0.4756	0.081*	
H8A2	0.0016	0.2891	0.3721	0.081*	
H8A3	0.176	0.3039	0.3895	0.081*	
C9A	−0.1868 (5)	0.17146 (9)	0.2339 (3)	0.0281 (11)	
H9A	−0.1931	0.1536	0.2704	0.034*	
C10A	−0.0589 (5)	0.16688 (11)	0.1779 (3)	0.0404 (13)	
H10A	−0.0914	0.1509	0.1343	0.061*	
H10B	0.0453	0.1624	0.2206	0.061*	
H10C	−0.0484	0.1844	0.1428	0.061*	
C11A	−0.3540 (5)	0.17688 (10)	0.1700 (3)	0.0396 (13)	
H11A	−0.3556	0.1958	0.1409	0.059*	
H11B	−0.4361	0.1762	0.2069	0.059*	
H11C	−0.3775	0.1621	0.1214	0.059*	
C12A	−0.2795 (5)	0.20298 (10)	0.3847 (3)	0.0304 (11)	
H12A	−0.3867	0.2059	0.34	0.036*	
C13A	−0.2494 (6)	0.22872 (12)	0.4501 (3)	0.0444 (14)	
H13A	−0.3357	0.23	0.4838	0.067*	
H13B	−0.2484	0.2463	0.4136	0.067*	
H13C	−0.1448	0.2265	0.4948	0.067*	
C14A	−0.2903 (6)	0.17527 (12)	0.4394 (4)	0.0522 (15)	
H14A	−0.188	0.1722	0.4855	0.078*	
H14B	−0.3113	0.159	0.3962	0.078*	
H14C	−0.3787	0.177	0.4715	0.078*	
C15A	−0.2766 (5)	0.27222 (9)	0.1602 (3)	0.0198 (10)	
Ni1A	−0.09895 (6)	0.241123 (11)	0.25206 (4)	0.01761 (14)	
O1A	−0.3167 (3)	0.24726 (6)	0.1911 (2)	0.0264 (7)	
O2A	−0.1191 (3)	0.27667 (6)	0.18928 (19)	0.0223 (7)	
O3A	−0.3730 (3)	0.28892 (6)	0.11296 (19)	0.0271 (7)	
P1A	−0.12663 (12)	0.20091 (2)	0.31662 (8)	0.0214 (3)	
P2A	0.15252 (12)	0.24007 (3)	0.32275 (8)	0.0228 (3)	
C1B	0.4161 (4)	0.03946 (9)	0.1585 (3)	0.0208 (10)	
H1B1	0.4405	0.0188	0.1704	0.025*	
H1B2	0.297	0.0419	0.144	0.025*	
C2B	0.4810 (4)	0.04938 (9)	0.0755 (3)	0.0201 (10)	
H2B1	0.4232	0.0669	0.0478	0.024*	
H2B2	0.4638	0.0342	0.0271	0.024*	
C3B	0.7525 (5)	0.07725 (9)	0.0237 (3)	0.0240 (10)	
H3B	0.7128	0.0666	−0.0362	0.029*	
C4B	0.6691 (6)	0.10641 (10)	0.0144 (3)	0.0397 (13)	
H4B1	0.6932	0.116	0.0753	0.06*	
H4B2	0.5519	0.1038	−0.0073	0.06*	
H4B3	0.7087	0.1182	−0.0307	0.06*	
C5B	0.9357 (5)	0.08070 (11)	0.0401 (3)	0.0389 (13)	
H5B1	0.9608	0.0925	−0.0097	0.058*	
H5B2	0.986	0.0618	0.0401	0.058*	
H5B3	0.9781	0.09	0.1006	0.058*	
C6B	0.7921 (4)	0.02135 (9)	0.1257 (3)	0.0187 (10)	
H6B	0.7227	0.0085	0.1542	0.022*	
C7B	0.7946 (5)	0.00803 (10)	0.0308 (3)	0.0270 (11)	
H7B1	0.8736	0.0182	0.0037	0.04*	
H7B2	0.6868	0.0096	−0.0112	0.04*	
H7B3	0.825	−0.0123	0.0393	0.04*	
C8B	0.9599 (4)	0.02111 (10)	0.1927 (3)	0.0289 (11)	
H8B1	0.9987	0.0013	0.2028	0.043*	
H8B2	0.9531	0.0296	0.2526	0.043*	
H8B3	1.0354	0.0323	0.1658	0.043*	
C9B	0.3595 (4)	0.08739 (10)	0.2764 (3)	0.0267 (11)	
H9B	0.2538	0.0774	0.2725	0.032*	
C10B	0.4088 (5)	0.10235 (11)	0.3715 (4)	0.0437 (14)	
H10D	0.515	0.1114	0.378	0.065*	
H10E	0.4146	0.0882	0.4213	0.065*	
H10F	0.3283	0.117	0.3759	0.065*	
C11B	0.3360 (5)	0.10932 (11)	0.1977 (4)	0.0439 (14)	
H11D	0.2513	0.123	0.2037	0.066*	
H11E	0.3038	0.0994	0.1374	0.066*	
H11F	0.4377	0.1197	0.2012	0.066*	
C12B	0.5298 (4)	0.03565 (10)	0.3619 (3)	0.0239 (10)	
H12B	0.5646	0.0473	0.4202	0.029*	
C13B	0.6622 (5)	0.01380 (10)	0.3618 (3)	0.0307 (11)	
H13D	0.6762	0.0016	0.4173	0.046*	
H13E	0.7639	0.0238	0.3625	0.046*	
H13F	0.632	0.0019	0.3055	0.046*	
C14B	0.3705 (5)	0.02115 (11)	0.3657 (3)	0.0383 (13)	
H14D	0.3359	0.0086	0.3113	0.057*	
H14E	0.2876	0.0358	0.3651	0.057*	
H14F	0.3857	0.0098	0.423	0.057*	
C15B	0.9336 (4)	0.10590 (9)	0.3390 (3)	0.0195 (10)	
Ni1B	0.73598 (5)	0.077729 (11)	0.24982 (4)	0.01499 (13)	
O1B	0.7989 (3)	0.09799 (6)	0.36375 (18)	0.0198 (7)	
O2B	0.9390 (3)	0.09517 (6)	0.25715 (19)	0.0204 (7)	
O3B	1.0375 (3)	0.12153 (6)	0.38621 (19)	0.0250 (7)	
P1B	0.50899 (11)	0.06037 (2)	0.26289 (7)	0.0180 (3)	
P2B	0.69677 (11)	0.05682 (2)	0.11706 (7)	0.0153 (2)	
C1C	0.0137 (4)	0.14384 (9)	0.6708 (3)	0.0212 (10)	
H1C1	−0.0619	0.1474	0.7115	0.025*	
H1C2	0.0272	0.162	0.6385	0.025*	
C2C	−0.0556 (4)	0.12052 (9)	0.5987 (3)	0.0229 (10)	
H2C1	−0.0037	0.1217	0.5453	0.027*	
H2C2	−0.1735	0.1234	0.5749	0.027*	
C3C	−0.0352 (4)	0.05938 (9)	0.5589 (3)	0.0188 (9)	
H3C	−0.1338	0.0641	0.5091	0.023*	
C4C	0.1129 (5)	0.06146 (10)	0.5169 (3)	0.0300 (11)	
H4C1	0.2098	0.0556	0.5636	0.045*	
H4C2	0.1257	0.0813	0.4978	0.045*	
H4C3	0.098	0.0488	0.4624	0.045*	
C5C	−0.0524 (5)	0.02856 (9)	0.5923 (3)	0.0237 (10)	
H5C1	−0.0582	0.0152	0.5402	0.036*	
H5C2	−0.1514	0.027	0.6152	0.036*	
H5C3	0.0415	0.0238	0.6428	0.036*	
C6C	−0.1881 (4)	0.08059 (10)	0.7094 (3)	0.0225 (10)	
H6C	−0.2004	0.0993	0.7402	0.027*	
C7C	−0.3499 (4)	0.07530 (10)	0.6393 (3)	0.0290 (11)	
H7C1	−0.3472	0.0566	0.6094	0.044*	
H7C2	−0.3682	0.0904	0.5916	0.044*	
H7C3	−0.4379	0.0756	0.672	0.044*	
C8C	−0.1566 (5)	0.05835 (10)	0.7871 (3)	0.0281 (11)	
H8C1	−0.2466	0.0583	0.8181	0.042*	
H8C2	−0.0558	0.063	0.8325	0.042*	
H8C3	−0.147	0.0393	0.7605	0.042*	
C9C	0.3637 (4)	0.14924 (9)	0.6907 (3)	0.0233 (10)	
H9C	0.3468	0.1705	0.6917	0.028*	
C10C	0.3441 (5)	0.14000 (11)	0.5898 (3)	0.0353 (12)	
H10G	0.4288	0.1489	0.5643	0.053*	
H10H	0.2376	0.146	0.5531	0.053*	
H10I	0.3533	0.1191	0.5869	0.053*	
C11C	0.5336 (4)	0.14270 (10)	0.7485 (3)	0.0286 (11)	
H11G	0.5498	0.1219	0.7526	0.043*	
H11H	0.5465	0.1507	0.8114	0.043*	
H11I	0.6137	0.1514	0.7187	0.043*	
C12C	0.2326 (5)	0.14893 (9)	0.8573 (3)	0.0241 (10)	
H12C	0.3496	0.1475	0.8901	0.029*	
C13C	0.1385 (6)	0.13196 (11)	0.9153 (3)	0.0457 (14)	
H13G	0.159	0.1401	0.9784	0.069*	
H13H	0.1734	0.1119	0.9188	0.069*	
H13I	0.0225	0.133	0.886	0.069*	
C14C	0.1893 (5)	0.18064 (10)	0.8518 (3)	0.0333 (12)	
H14G	0.073	0.1829	0.8248	0.05*	
H14H	0.2504	0.1905	0.8125	0.05*	
H14I	0.2166	0.1889	0.9147	0.05*	
C15C	0.3927 (4)	0.05090 (9)	0.8190 (3)	0.0196 (10)	
Ni1C	0.21570 (5)	0.085830 (11)	0.74854 (4)	0.01691 (13)	
O1C	0.4212 (3)	0.07890 (6)	0.82835 (19)	0.0234 (7)	
O2C	0.2466 (3)	0.04613 (6)	0.76615 (19)	0.0220 (7)	
O3C	0.4888 (3)	0.03226 (6)	0.85383 (19)	0.0231 (7)	
P1C	0.21058 (12)	0.13190 (2)	0.74185 (8)	0.0189 (3)	
P2C	−0.01785 (11)	0.08543 (2)	0.65341 (7)	0.0171 (2)	

### Atomic displacement parameters (Å^2^)

**Table:**

	*U*^11^	*U*^22^	*U*^33^	*U*^12^	*U*^13^	*U*^23^
C1A	0.030 (2)	0.016 (3)	0.028 (3)	0.0080 (19)	−0.0017 (19)	0.005 (2)
C2A	0.026 (2)	0.030 (3)	0.035 (3)	0.009 (2)	−0.001 (2)	0.006 (2)
C3A	0.018 (2)	0.035 (3)	0.034 (3)	−0.001 (2)	0.0039 (19)	−0.001 (2)
C4A	0.034 (3)	0.041 (3)	0.037 (3)	−0.001 (2)	0.015 (2)	−0.003 (3)
C5A	0.038 (3)	0.047 (4)	0.047 (4)	−0.008 (2)	0.016 (2)	0.012 (3)
C6A	0.027 (2)	0.035 (3)	0.034 (3)	−0.004 (2)	0.000 (2)	−0.010 (2)
C7A	0.032 (3)	0.054 (4)	0.042 (3)	−0.004 (2)	−0.005 (2)	−0.014 (3)
C8A	0.061 (3)	0.046 (4)	0.046 (4)	0.009 (3)	−0.006 (3)	−0.017 (3)
C9A	0.035 (2)	0.011 (2)	0.033 (3)	−0.0028 (19)	−0.004 (2)	0.002 (2)
C10A	0.050 (3)	0.029 (3)	0.039 (3)	0.003 (2)	0.004 (2)	−0.014 (2)
C11A	0.041 (3)	0.022 (3)	0.045 (3)	0.001 (2)	−0.011 (2)	−0.003 (2)
C12A	0.034 (2)	0.026 (3)	0.033 (3)	−0.003 (2)	0.011 (2)	0.007 (2)
C13A	0.048 (3)	0.052 (4)	0.039 (3)	0.019 (3)	0.021 (2)	0.005 (3)
C14A	0.054 (3)	0.049 (4)	0.060 (4)	0.001 (3)	0.027 (3)	0.025 (3)
C15A	0.031 (2)	0.014 (2)	0.013 (2)	0.0052 (19)	0.0028 (18)	−0.0026 (19)
Ni1A	0.0189 (3)	0.0126 (3)	0.0195 (3)	0.0011 (2)	0.0009 (2)	0.0015 (2)
O1A	0.0207 (15)	0.0216 (18)	0.0339 (19)	−0.0012 (13)	0.0005 (12)	0.0099 (15)
O2A	0.0219 (15)	0.0147 (17)	0.0279 (18)	0.0003 (12)	0.0011 (12)	0.0043 (13)
O3A	0.0320 (16)	0.0214 (18)	0.0234 (18)	0.0081 (14)	−0.0024 (13)	0.0046 (14)
P1A	0.0227 (6)	0.0149 (6)	0.0240 (7)	0.0013 (5)	0.0001 (5)	0.0031 (5)
P2A	0.0190 (5)	0.0212 (7)	0.0257 (7)	−0.0001 (5)	0.0004 (5)	0.0001 (5)
C1B	0.0146 (19)	0.024 (3)	0.021 (3)	−0.0017 (18)	0.0000 (17)	−0.003 (2)
C2B	0.0146 (19)	0.027 (3)	0.018 (2)	0.0008 (18)	0.0021 (16)	−0.004 (2)
C3B	0.036 (2)	0.023 (3)	0.014 (2)	−0.010 (2)	0.0070 (18)	0.000 (2)
C4B	0.064 (3)	0.022 (3)	0.031 (3)	−0.012 (2)	0.007 (2)	0.006 (2)
C5B	0.045 (3)	0.049 (4)	0.027 (3)	−0.024 (3)	0.018 (2)	0.002 (2)
C6B	0.018 (2)	0.014 (2)	0.023 (3)	−0.0004 (17)	0.0038 (17)	0.0015 (19)
C7B	0.024 (2)	0.025 (3)	0.031 (3)	0.0029 (19)	0.0052 (19)	−0.011 (2)
C8B	0.023 (2)	0.029 (3)	0.032 (3)	0.002 (2)	0.0027 (19)	−0.001 (2)
C9B	0.019 (2)	0.032 (3)	0.031 (3)	0.000 (2)	0.0095 (18)	−0.011 (2)
C10B	0.027 (2)	0.049 (4)	0.056 (4)	0.003 (2)	0.012 (2)	−0.027 (3)
C11B	0.036 (3)	0.034 (3)	0.062 (4)	0.013 (2)	0.012 (2)	−0.002 (3)
C12B	0.023 (2)	0.032 (3)	0.018 (3)	−0.010 (2)	0.0069 (18)	−0.005 (2)
C13B	0.037 (3)	0.034 (3)	0.022 (3)	−0.003 (2)	0.008 (2)	0.007 (2)
C14B	0.034 (3)	0.053 (4)	0.030 (3)	−0.011 (2)	0.012 (2)	0.008 (3)
C15B	0.023 (2)	0.013 (2)	0.018 (3)	−0.0016 (18)	−0.0032 (18)	0.0047 (19)
Ni1B	0.0141 (2)	0.0162 (3)	0.0144 (3)	−0.0024 (2)	0.0028 (2)	−0.0025 (2)
O1B	0.0186 (14)	0.0237 (17)	0.0173 (17)	−0.0043 (12)	0.0044 (12)	−0.0058 (13)
O2B	0.0173 (14)	0.0265 (18)	0.0169 (17)	−0.0090 (12)	0.0031 (11)	−0.0040 (14)
O3B	0.0250 (15)	0.0214 (18)	0.0246 (18)	−0.0047 (13)	−0.0021 (12)	−0.0014 (14)
P1B	0.0153 (5)	0.0207 (6)	0.0184 (7)	−0.0013 (5)	0.0047 (4)	−0.0047 (5)
P2B	0.0162 (5)	0.0151 (6)	0.0145 (6)	−0.0025 (4)	0.0036 (4)	−0.0006 (5)
C1C	0.025 (2)	0.013 (2)	0.028 (3)	0.0046 (18)	0.0096 (18)	0.009 (2)
C2C	0.020 (2)	0.022 (3)	0.025 (3)	0.0040 (19)	0.0026 (18)	0.001 (2)
C3C	0.020 (2)	0.021 (3)	0.015 (2)	0.0029 (18)	0.0052 (17)	−0.0022 (19)
C4C	0.041 (3)	0.029 (3)	0.023 (3)	0.006 (2)	0.014 (2)	0.002 (2)
C5C	0.026 (2)	0.022 (3)	0.024 (3)	0.0001 (19)	0.0064 (18)	−0.007 (2)
C6C	0.020 (2)	0.027 (3)	0.022 (3)	0.0052 (19)	0.0075 (17)	−0.005 (2)
C7C	0.023 (2)	0.030 (3)	0.035 (3)	−0.002 (2)	0.0091 (19)	−0.004 (2)
C8C	0.033 (2)	0.031 (3)	0.023 (3)	−0.004 (2)	0.013 (2)	0.001 (2)
C9C	0.027 (2)	0.013 (2)	0.032 (3)	−0.0003 (18)	0.0121 (19)	0.004 (2)
C10C	0.034 (3)	0.044 (3)	0.032 (3)	0.001 (2)	0.015 (2)	0.012 (2)
C11C	0.021 (2)	0.028 (3)	0.037 (3)	−0.004 (2)	0.0090 (19)	−0.004 (2)
C12C	0.032 (2)	0.019 (3)	0.023 (3)	−0.0006 (19)	0.0099 (19)	0.000 (2)
C13C	0.074 (4)	0.034 (3)	0.040 (4)	−0.012 (3)	0.034 (3)	−0.006 (3)
C14C	0.044 (3)	0.023 (3)	0.034 (3)	−0.001 (2)	0.010 (2)	−0.008 (2)
C15C	0.019 (2)	0.024 (3)	0.017 (3)	−0.002 (2)	0.0054 (18)	−0.004 (2)
Ni1C	0.0175 (3)	0.0123 (3)	0.0202 (3)	0.0011 (2)	0.0029 (2)	0.0022 (2)
O1C	0.0202 (14)	0.0162 (17)	0.0295 (19)	−0.0020 (12)	−0.0030 (12)	−0.0007 (14)
O2C	0.0171 (14)	0.0165 (16)	0.0277 (18)	0.0028 (12)	−0.0039 (12)	0.0004 (13)
O3C	0.0216 (14)	0.0179 (17)	0.0268 (18)	0.0047 (13)	−0.0004 (12)	0.0065 (14)
P1C	0.0198 (5)	0.0145 (6)	0.0228 (7)	0.0012 (5)	0.0061 (4)	0.0027 (5)
P2C	0.0183 (5)	0.0142 (6)	0.0185 (6)	0.0021 (5)	0.0039 (4)	0.0007 (5)

### Geometric parameters (Å, º)

**Table:**

C1A—C2A	1.537 (6)	C9B—C10B	1.532 (6)
C1A—P1A	1.825 (4)	C9B—P1B	1.832 (4)
C1A—H1A1	0.99	C9B—H9B	1
C1A—H1A2	0.99	C10B—H10D	0.98
C2A—P2A	1.833 (4)	C10B—H10E	0.98
C2A—H2A1	0.99	C10B—H10F	0.98
C2A—H2A2	0.99	C11B—H11D	0.98
C3A—C5A	1.509 (6)	C11B—H11E	0.98
C3A—C4A	1.545 (6)	C11B—H11F	0.98
C3A—P2A	1.837 (4)	C12B—C13B	1.517 (6)
C3A—H3A	1	C12B—C14B	1.525 (5)
C4A—H4A1	0.98	C12B—P1B	1.834 (4)
C4A—H4A2	0.98	C12B—H12B	1
C4A—H4A3	0.98	C13B—H13D	0.98
C5A—H5A1	0.98	C13B—H13E	0.98
C5A—H5A2	0.98	C13B—H13F	0.98
C5A—H5A3	0.98	C14B—H14D	0.98
C6A—C8A	1.501 (6)	C14B—H14E	0.98
C6A—C7A	1.530 (5)	C14B—H14F	0.98
C6A—P2A	1.827 (5)	C15B—O3B	1.227 (4)
C6A—H6A	1	C15B—O2B	1.316 (5)
C7A—H7A1	0.98	C15B—O1B	1.332 (4)
C7A—H7A2	0.98	C15B—Ni1B	2.287 (4)
C7A—H7A3	0.98	Ni1B—O2B	1.887 (2)
C8A—H8A1	0.98	Ni1B—O1B	1.890 (3)
C8A—H8A2	0.98	Ni1B—P2B	2.1399 (12)
C8A—H8A3	0.98	Ni1B—P1B	2.1415 (11)
C9A—C10A	1.526 (6)	C1C—C2C	1.536 (6)
C9A—C11A	1.529 (5)	C1C—P1C	1.838 (4)
C9A—P1A	1.826 (4)	C1C—H1C1	0.99
C9A—H9A	1	C1C—H1C2	0.99
C10A—H10A	0.98	C2C—P2C	1.817 (4)
C10A—H10B	0.98	C2C—H2C1	0.99
C10A—H10C	0.98	C2C—H2C2	0.99
C11A—H11A	0.98	C3C—C4C	1.531 (5)
C11A—H11B	0.98	C3C—C5C	1.536 (6)
C11A—H11C	0.98	C3C—P2C	1.827 (4)
C12A—C13A	1.521 (7)	C3C—H3C	1
C12A—C14A	1.536 (6)	C4C—H4C1	0.98
C12A—P1A	1.821 (4)	C4C—H4C2	0.98
C12A—H12A	1	C4C—H4C3	0.98
C13A—H13A	0.98	C5C—H5C1	0.98
C13A—H13B	0.98	C5C—H5C2	0.98
C13A—H13C	0.98	C5C—H5C3	0.98
C14A—H14A	0.98	C6C—C8C	1.520 (6)
C14A—H14B	0.98	C6C—C7C	1.533 (5)
C14A—H14C	0.98	C6C—P2C	1.841 (4)
C15A—O3A	1.221 (4)	C6C—H6C	1
C15A—O2A	1.322 (4)	C7C—H7C1	0.98
C15A—O1A	1.322 (5)	C7C—H7C2	0.98
C15A—Ni1A	2.291 (4)	C7C—H7C3	0.98
Ni1A—O1A	1.879 (2)	C8C—H8C1	0.98
Ni1A—O2A	1.885 (3)	C8C—H8C2	0.98
Ni1A—P1A	2.1390 (12)	C8C—H8C3	0.98
Ni1A—P2A	2.1460 (11)	C9C—C10C	1.520 (6)
C1B—C2B	1.526 (5)	C9C—C11C	1.524 (5)
C1B—P1B	1.835 (4)	C9C—P1C	1.837 (4)
C1B—H1B1	0.99	C9C—H9C	1
C1B—H1B2	0.99	C10C—H10G	0.98
C2B—P2B	1.825 (3)	C10C—H10H	0.98
C2B—H2B1	0.99	C10C—H10I	0.98
C2B—H2B2	0.99	C11C—H11G	0.98
C3B—C4B	1.523 (6)	C11C—H11H	0.98
C3B—C5B	1.527 (5)	C11C—H11I	0.98
C3B—P2B	1.825 (4)	C12C—C14C	1.520 (6)
C3B—H3B	1	C12C—C13C	1.522 (6)
C4B—H4B1	0.98	C12C—P1C	1.847 (4)
C4B—H4B2	0.98	C12C—H12C	1
C4B—H4B3	0.98	C13C—H13G	0.98
C5B—H5B1	0.98	C13C—H13H	0.98
C5B—H5B2	0.98	C13C—H13I	0.98
C5B—H5B3	0.98	C14C—H14G	0.98
C6B—C8B	1.532 (5)	C14C—H14H	0.98
C6B—C7B	1.535 (5)	C14C—H14I	0.98
C6B—P2B	1.831 (4)	C15C—O3C	1.220 (4)
C6B—H6B	1	C15C—O2C	1.320 (4)
C7B—H7B1	0.98	C15C—O1C	1.328 (5)
C7B—H7B2	0.98	C15C—Ni1C	2.291 (4)
C7B—H7B3	0.98	Ni1C—O2C	1.877 (3)
C8B—H8B1	0.98	Ni1C—O1C	1.889 (2)
C8B—H8B2	0.98	Ni1C—P2C	2.1433 (10)
C8B—H8B3	0.98	Ni1C—P1C	2.1481 (12)
C9B—C11B	1.523 (6)		
			
C2A—C1A—P1A	110.0 (3)	C9B—C10B—H10F	109.5
C2A—C1A—H1A1	109.7	H10D—C10B—H10F	109.5
P1A—C1A—H1A1	109.7	H10E—C10B—H10F	109.5
C2A—C1A—H1A2	109.7	C9B—C11B—H11D	109.5
P1A—C1A—H1A2	109.7	C9B—C11B—H11E	109.5
H1A1—C1A—H1A2	108.2	H11D—C11B—H11E	109.5
C1A—C2A—P2A	109.4 (3)	C9B—C11B—H11F	109.5
C1A—C2A—H2A1	109.8	H11D—C11B—H11F	109.5
P2A—C2A—H2A1	109.8	H11E—C11B—H11F	109.5
C1A—C2A—H2A2	109.8	C13B—C12B—C14B	111.6 (4)
P2A—C2A—H2A2	109.8	C13B—C12B—P1B	110.7 (3)
H2A1—C2A—H2A2	108.2	C14B—C12B—P1B	112.8 (3)
C5A—C3A—C4A	110.9 (4)	C13B—C12B—H12B	107.2
C5A—C3A—P2A	112.6 (3)	C14B—C12B—H12B	107.2
C4A—C3A—P2A	109.7 (3)	P1B—C12B—H12B	107.2
C5A—C3A—H3A	107.8	C12B—C13B—H13D	109.5
C4A—C3A—H3A	107.8	C12B—C13B—H13E	109.5
P2A—C3A—H3A	107.8	H13D—C13B—H13E	109.5
C3A—C4A—H4A1	109.5	C12B—C13B—H13F	109.5
C3A—C4A—H4A2	109.5	H13D—C13B—H13F	109.5
H4A1—C4A—H4A2	109.5	H13E—C13B—H13F	109.5
C3A—C4A—H4A3	109.5	C12B—C14B—H14D	109.5
H4A1—C4A—H4A3	109.5	C12B—C14B—H14E	109.5
H4A2—C4A—H4A3	109.5	H14D—C14B—H14E	109.5
C3A—C5A—H5A1	109.5	C12B—C14B—H14F	109.5
C3A—C5A—H5A2	109.5	H14D—C14B—H14F	109.5
H5A1—C5A—H5A2	109.5	H14E—C14B—H14F	109.5
C3A—C5A—H5A3	109.5	O3B—C15B—O2B	124.7 (4)
H5A1—C5A—H5A3	109.5	O3B—C15B—O1B	124.0 (4)
H5A2—C5A—H5A3	109.5	O2B—C15B—O1B	111.3 (3)
C8A—C6A—C7A	111.0 (4)	O3B—C15B—Ni1B	178.6 (3)
C8A—C6A—P2A	113.8 (3)	O2B—C15B—Ni1B	55.59 (18)
C7A—C6A—P2A	114.8 (3)	O1B—C15B—Ni1B	55.71 (18)
C8A—C6A—H6A	105.4	O2B—Ni1B—O1B	70.75 (11)
C7A—C6A—H6A	105.4	O2B—Ni1B—P2B	101.06 (9)
P2A—C6A—H6A	105.4	O1B—Ni1B—P2B	171.81 (8)
C6A—C7A—H7A1	109.5	O2B—Ni1B—P1B	171.17 (9)
C6A—C7A—H7A2	109.5	O1B—Ni1B—P1B	100.44 (8)
H7A1—C7A—H7A2	109.5	P2B—Ni1B—P1B	87.75 (4)
C6A—C7A—H7A3	109.5	O2B—Ni1B—C15B	35.14 (13)
H7A1—C7A—H7A3	109.5	O1B—Ni1B—C15B	35.62 (13)
H7A2—C7A—H7A3	109.5	P2B—Ni1B—C15B	136.19 (11)
C6A—C8A—H8A1	109.5	P1B—Ni1B—C15B	136.06 (12)
C6A—C8A—H8A2	109.5	C15B—O1B—Ni1B	88.7 (2)
H8A1—C8A—H8A2	109.5	C15B—O2B—Ni1B	89.3 (2)
C6A—C8A—H8A3	109.5	C9B—P1B—C12B	106.5 (2)
H8A1—C8A—H8A3	109.5	C9B—P1B—C1B	106.47 (18)
H8A2—C8A—H8A3	109.5	C12B—P1B—C1B	106.0 (2)
C10A—C9A—C11A	111.6 (4)	C9B—P1B—Ni1B	114.38 (15)
C10A—C9A—P1A	110.0 (3)	C12B—P1B—Ni1B	112.86 (12)
C11A—C9A—P1A	111.1 (3)	C1B—P1B—Ni1B	110.11 (13)
C10A—C9A—H9A	108	C2B—P2B—C3B	105.38 (18)
C11A—C9A—H9A	108	C2B—P2B—C6B	104.17 (18)
P1A—C9A—H9A	108	C3B—P2B—C6B	109.5 (2)
C9A—C10A—H10A	109.5	C2B—P2B—Ni1B	108.71 (14)
C9A—C10A—H10B	109.5	C3B—P2B—Ni1B	116.09 (15)
H10A—C10A—H10B	109.5	C6B—P2B—Ni1B	112.08 (13)
C9A—C10A—H10C	109.5	C2C—C1C—P1C	109.2 (3)
H10A—C10A—H10C	109.5	C2C—C1C—H1C1	109.8
H10B—C10A—H10C	109.5	P1C—C1C—H1C1	109.8
C9A—C11A—H11A	109.5	C2C—C1C—H1C2	109.8
C9A—C11A—H11B	109.5	P1C—C1C—H1C2	109.8
H11A—C11A—H11B	109.5	H1C1—C1C—H1C2	108.3
C9A—C11A—H11C	109.5	C1C—C2C—P2C	109.3 (3)
H11A—C11A—H11C	109.5	C1C—C2C—H2C1	109.8
H11B—C11A—H11C	109.5	P2C—C2C—H2C1	109.8
C13A—C12A—C14A	110.6 (4)	C1C—C2C—H2C2	109.8
C13A—C12A—P1A	110.8 (3)	P2C—C2C—H2C2	109.8
C14A—C12A—P1A	112.5 (3)	H2C1—C2C—H2C2	108.3
C13A—C12A—H12A	107.5	C4C—C3C—C5C	109.7 (3)
C14A—C12A—H12A	107.5	C4C—C3C—P2C	109.8 (3)
P1A—C12A—H12A	107.5	C5C—C3C—P2C	111.9 (3)
C12A—C13A—H13A	109.5	C4C—C3C—H3C	108.4
C12A—C13A—H13B	109.5	C5C—C3C—H3C	108.4
H13A—C13A—H13B	109.5	P2C—C3C—H3C	108.4
C12A—C13A—H13C	109.5	C3C—C4C—H4C1	109.5
H13A—C13A—H13C	109.5	C3C—C4C—H4C2	109.5
H13B—C13A—H13C	109.5	H4C1—C4C—H4C2	109.5
C12A—C14A—H14A	109.5	C3C—C4C—H4C3	109.5
C12A—C14A—H14B	109.5	H4C1—C4C—H4C3	109.5
H14A—C14A—H14B	109.5	H4C2—C4C—H4C3	109.5
C12A—C14A—H14C	109.5	C3C—C5C—H5C1	109.5
H14A—C14A—H14C	109.5	C3C—C5C—H5C2	109.5
H14B—C14A—H14C	109.5	H5C1—C5C—H5C2	109.5
O3A—C15A—O2A	125.2 (4)	C3C—C5C—H5C3	109.5
O3A—C15A—O1A	124.3 (4)	H5C1—C5C—H5C3	109.5
O2A—C15A—O1A	110.5 (3)	H5C2—C5C—H5C3	109.5
O3A—C15A—Ni1A	178.5 (3)	C8C—C6C—C7C	111.7 (4)
O2A—C15A—Ni1A	55.36 (18)	C8C—C6C—P2C	113.8 (3)
O1A—C15A—Ni1A	55.10 (18)	C7C—C6C—P2C	113.1 (3)
O1A—Ni1A—O2A	70.50 (11)	C8C—C6C—H6C	105.8
O1A—Ni1A—P1A	98.44 (9)	C7C—C6C—H6C	105.8
O2A—Ni1A—P1A	168.79 (8)	P2C—C6C—H6C	105.8
O1A—Ni1A—P2A	172.56 (10)	C6C—C7C—H7C1	109.5
O2A—Ni1A—P2A	102.91 (8)	C6C—C7C—H7C2	109.5
P1A—Ni1A—P2A	88.01 (4)	H7C1—C7C—H7C2	109.5
O1A—Ni1A—C15A	35.25 (13)	C6C—C7C—H7C3	109.5
O2A—Ni1A—C15A	35.25 (12)	H7C1—C7C—H7C3	109.5
P1A—Ni1A—C15A	133.66 (11)	H7C2—C7C—H7C3	109.5
P2A—Ni1A—C15A	138.05 (11)	C6C—C8C—H8C1	109.5
C15A—O1A—Ni1A	89.7 (2)	C6C—C8C—H8C2	109.5
C15A—O2A—Ni1A	89.4 (2)	H8C1—C8C—H8C2	109.5
C12A—P1A—C1A	106.8 (2)	C6C—C8C—H8C3	109.5
C12A—P1A—C9A	106.5 (2)	H8C1—C8C—H8C3	109.5
C1A—P1A—C9A	106.93 (19)	H8C2—C8C—H8C3	109.5
C12A—P1A—Ni1A	111.46 (15)	C10C—C9C—C11C	111.2 (3)
C1A—P1A—Ni1A	110.94 (14)	C10C—C9C—P1C	110.6 (3)
C9A—P1A—Ni1A	113.81 (16)	C11C—C9C—P1C	110.6 (3)
C6A—P2A—C2A	103.1 (2)	C10C—C9C—H9C	108.1
C6A—P2A—C3A	109.8 (2)	C11C—C9C—H9C	108.1
C2A—P2A—C3A	106.0 (2)	P1C—C9C—H9C	108.1
C6A—P2A—Ni1A	110.17 (14)	C9C—C10C—H10G	109.5
C2A—P2A—Ni1A	109.42 (14)	C9C—C10C—H10H	109.5
C3A—P2A—Ni1A	117.29 (15)	H10G—C10C—H10H	109.5
C2B—C1B—P1B	110.2 (3)	C9C—C10C—H10I	109.5
C2B—C1B—H1B1	109.6	H10G—C10C—H10I	109.5
P1B—C1B—H1B1	109.6	H10H—C10C—H10I	109.5
C2B—C1B—H1B2	109.6	C9C—C11C—H11G	109.5
P1B—C1B—H1B2	109.6	C9C—C11C—H11H	109.5
H1B1—C1B—H1B2	108.1	H11G—C11C—H11H	109.5
C1B—C2B—P2B	108.3 (3)	C9C—C11C—H11I	109.5
C1B—C2B—H2B1	110	H11G—C11C—H11I	109.5
P2B—C2B—H2B1	110	H11H—C11C—H11I	109.5
C1B—C2B—H2B2	110	C14C—C12C—C13C	112.5 (4)
P2B—C2B—H2B2	110	C14C—C12C—P1C	113.4 (3)
H2B1—C2B—H2B2	108.4	C13C—C12C—P1C	110.0 (3)
C4B—C3B—C5B	110.8 (4)	C14C—C12C—H12C	106.8
C4B—C3B—P2B	109.6 (3)	C13C—C12C—H12C	106.8
C5B—C3B—P2B	111.8 (3)	P1C—C12C—H12C	106.8
C4B—C3B—H3B	108.2	C12C—C13C—H13G	109.5
C5B—C3B—H3B	108.2	C12C—C13C—H13H	109.5
P2B—C3B—H3B	108.2	H13G—C13C—H13H	109.5
C3B—C4B—H4B1	109.5	C12C—C13C—H13I	109.5
C3B—C4B—H4B2	109.5	H13G—C13C—H13I	109.5
H4B1—C4B—H4B2	109.5	H13H—C13C—H13I	109.5
C3B—C4B—H4B3	109.5	C12C—C14C—H14G	109.5
H4B1—C4B—H4B3	109.5	C12C—C14C—H14H	109.5
H4B2—C4B—H4B3	109.5	H14G—C14C—H14H	109.5
C3B—C5B—H5B1	109.5	C12C—C14C—H14I	109.5
C3B—C5B—H5B2	109.5	H14G—C14C—H14I	109.5
H5B1—C5B—H5B2	109.5	H14H—C14C—H14I	109.5
C3B—C5B—H5B3	109.5	O3C—C15C—O2C	124.9 (4)
H5B1—C5B—H5B3	109.5	O3C—C15C—O1C	124.6 (3)
H5B2—C5B—H5B3	109.5	O2C—C15C—O1C	110.5 (3)
C8B—C6B—C7B	112.0 (3)	O3C—C15C—Ni1C	177.9 (3)
C8B—C6B—P2B	112.4 (3)	O2C—C15C—Ni1C	55.01 (19)
C7B—C6B—P2B	113.7 (3)	O1C—C15C—Ni1C	55.54 (19)
C8B—C6B—H6B	106	O2C—Ni1C—O1C	70.55 (11)
C7B—C6B—H6B	106	O2C—Ni1C—P2C	99.18 (8)
P2B—C6B—H6B	106	O1C—Ni1C—P2C	169.54 (9)
C6B—C7B—H7B1	109.5	O2C—Ni1C—P1C	172.35 (8)
C6B—C7B—H7B2	109.5	O1C—Ni1C—P1C	101.83 (9)
H7B1—C7B—H7B2	109.5	P2C—Ni1C—P1C	88.46 (4)
C6B—C7B—H7B3	109.5	O2C—Ni1C—C15C	35.16 (12)
H7B1—C7B—H7B3	109.5	O1C—Ni1C—C15C	35.40 (12)
H7B2—C7B—H7B3	109.5	P2C—Ni1C—C15C	134.25 (11)
C6B—C8B—H8B1	109.5	P1C—Ni1C—C15C	137.23 (11)
C6B—C8B—H8B2	109.5	C15C—O1C—Ni1C	89.1 (2)
H8B1—C8B—H8B2	109.5	C15C—O2C—Ni1C	89.8 (2)
C6B—C8B—H8B3	109.5	C9C—P1C—C1C	105.72 (19)
H8B1—C8B—H8B3	109.5	C9C—P1C—C12C	105.13 (19)
H8B2—C8B—H8B3	109.5	C1C—P1C—C12C	106.01 (19)
C11B—C9B—C10B	110.5 (4)	C9C—P1C—Ni1C	116.71 (14)
C11B—C9B—P1B	110.3 (3)	C1C—P1C—Ni1C	109.53 (14)
C10B—C9B—P1B	111.4 (3)	C12C—P1C—Ni1C	112.98 (14)
C11B—C9B—H9B	108.2	C2C—P2C—C3C	106.6 (2)
C10B—C9B—H9B	108.2	C2C—P2C—C6C	103.51 (19)
P1B—C9B—H9B	108.2	C3C—P2C—C6C	109.25 (18)
C9B—C10B—H10D	109.5	C2C—P2C—Ni1C	108.77 (13)
C9B—C10B—H10E	109.5	C3C—P2C—Ni1C	113.61 (13)
H10D—C10B—H10E	109.5	C6C—P2C—Ni1C	114.35 (13)
			
P1A—C1A—C2A—P2A	37.1 (4)	P2B—Ni1B—P1B—C9B	−120.77 (16)
O3A—C15A—Ni1A—O1A	68 (13)	C15B—Ni1B—P1B—C9B	59.6 (2)
O2A—C15A—Ni1A—O1A	−179.5 (4)	O2B—Ni1B—P1B—C12B	−58.2 (6)
O3A—C15A—Ni1A—O2A	−112 (13)	O1B—Ni1B—P1B—C12B	−62.57 (18)
O1A—C15A—Ni1A—O2A	179.5 (4)	P2B—Ni1B—P1B—C12B	117.26 (16)
O3A—C15A—Ni1A—P1A	65 (13)	C15B—Ni1B—P1B—C12B	−62.4 (2)
O2A—C15A—Ni1A—P1A	177.52 (17)	O2B—Ni1B—P1B—C1B	−176.4 (6)
O1A—C15A—Ni1A—P1A	−2.9 (3)	O1B—Ni1B—P1B—C1B	179.23 (17)
O3A—C15A—Ni1A—P2A	−106 (13)	P2B—Ni1B—P1B—C1B	−0.95 (15)
O2A—C15A—Ni1A—P2A	5.7 (3)	C15B—Ni1B—P1B—C1B	179.4 (2)
O1A—C15A—Ni1A—P2A	−174.78 (18)	C1B—C2B—P2B—C3B	−165.3 (3)
O3A—C15A—O1A—Ni1A	−178.3 (4)	C1B—C2B—P2B—C6B	79.5 (3)
O2A—C15A—O1A—Ni1A	0.4 (3)	C1B—C2B—P2B—Ni1B	−40.2 (3)
O2A—Ni1A—O1A—C15A	−0.3 (2)	C4B—C3B—P2B—C2B	66.7 (3)
P1A—Ni1A—O1A—C15A	177.8 (2)	C5B—C3B—P2B—C2B	−170.0 (3)
P2A—Ni1A—O1A—C15A	28.0 (9)	C4B—C3B—P2B—C6B	178.3 (3)
O3A—C15A—O2A—Ni1A	178.3 (4)	C5B—C3B—P2B—C6B	−58.5 (4)
O1A—C15A—O2A—Ni1A	−0.4 (3)	C4B—C3B—P2B—Ni1B	−53.6 (3)
O1A—Ni1A—O2A—C15A	0.3 (2)	C5B—C3B—P2B—Ni1B	69.7 (3)
P1A—Ni1A—O2A—C15A	−9.3 (6)	C8B—C6B—P2B—C2B	−158.0 (3)
P2A—Ni1A—O2A—C15A	−176.1 (2)	C7B—C6B—P2B—C2B	73.5 (3)
C13A—C12A—P1A—C1A	71.3 (4)	C8B—C6B—P2B—C3B	89.7 (3)
C14A—C12A—P1A—C1A	−53.2 (4)	C7B—C6B—P2B—C3B	−38.8 (3)
C13A—C12A—P1A—C9A	−174.7 (3)	C8B—C6B—P2B—Ni1B	−40.6 (3)
C14A—C12A—P1A—C9A	60.8 (4)	C7B—C6B—P2B—Ni1B	−169.2 (2)
C13A—C12A—P1A—Ni1A	−50.1 (4)	O2B—Ni1B—P2B—C2B	−159.86 (17)
C14A—C12A—P1A—Ni1A	−174.5 (3)	O1B—Ni1B—P2B—C2B	−160.4 (6)
C2A—C1A—P1A—C12A	−147.5 (3)	P1B—Ni1B—P2B—C2B	20.84 (15)
C2A—C1A—P1A—C9A	98.8 (3)	C15B—Ni1B—P2B—C2B	−159.5 (2)
C2A—C1A—P1A—Ni1A	−25.9 (3)	O2B—Ni1B—P2B—C3B	−41.33 (17)
C10A—C9A—P1A—C12A	−176.3 (3)	O1B—Ni1B—P2B—C3B	−41.8 (7)
C11A—C9A—P1A—C12A	59.6 (4)	P1B—Ni1B—P2B—C3B	139.38 (15)
C10A—C9A—P1A—C1A	−62.3 (4)	C15B—Ni1B—P2B—C3B	−41.0 (2)
C11A—C9A—P1A—C1A	173.6 (3)	O2B—Ni1B—P2B—C6B	85.53 (16)
C10A—C9A—P1A—Ni1A	60.5 (3)	O1B—Ni1B—P2B—C6B	85.0 (7)
C11A—C9A—P1A—Ni1A	−63.6 (3)	P1B—Ni1B—P2B—C6B	−93.76 (14)
O1A—Ni1A—P1A—C12A	−53.44 (19)	C15B—Ni1B—P2B—C6B	85.9 (2)
O2A—Ni1A—P1A—C12A	−44.3 (5)	P1C—C1C—C2C—P2C	40.7 (3)
P2A—Ni1A—P1A—C12A	122.83 (16)	O3C—C15C—Ni1C—O2C	89 (9)
C15A—Ni1A—P1A—C12A	−51.7 (2)	O1C—C15C—Ni1C—O2C	−177.6 (4)
O1A—Ni1A—P1A—C1A	−172.34 (19)	O3C—C15C—Ni1C—O1C	−94 (9)
O2A—Ni1A—P1A—C1A	−163.2 (5)	O2C—C15C—Ni1C—O1C	177.6 (4)
P2A—Ni1A—P1A—C1A	3.92 (17)	O3C—C15C—Ni1C—P2C	84 (9)
C15A—Ni1A—P1A—C1A	−170.6 (2)	O2C—C15C—Ni1C—P2C	−4.9 (3)
O1A—Ni1A—P1A—C9A	67.01 (17)	O1C—C15C—Ni1C—P2C	177.53 (16)
O2A—Ni1A—P1A—C9A	76.1 (5)	O3C—C15C—Ni1C—P1C	−93 (9)
P2A—Ni1A—P1A—C9A	−116.72 (15)	O2C—C15C—Ni1C—P1C	178.66 (16)
C15A—Ni1A—P1A—C9A	68.7 (2)	O1C—C15C—Ni1C—P1C	1.1 (3)
C8A—C6A—P2A—C2A	−163.5 (4)	O3C—C15C—O1C—Ni1C	177.4 (4)
C7A—C6A—P2A—C2A	67.0 (4)	O2C—C15C—O1C—Ni1C	−2.1 (3)
C8A—C6A—P2A—C3A	83.8 (4)	O2C—Ni1C—O1C—C15C	1.5 (2)
C7A—C6A—P2A—C3A	−45.6 (4)	P2C—Ni1C—O1C—C15C	−9.8 (6)
C8A—C6A—P2A—Ni1A	−46.8 (4)	P1C—Ni1C—O1C—C15C	−179.3 (2)
C7A—C6A—P2A—Ni1A	−176.3 (3)	O3C—C15C—O2C—Ni1C	−177.4 (4)
C1A—C2A—P2A—C6A	82.8 (3)	O1C—C15C—O2C—Ni1C	2.1 (3)
C1A—C2A—P2A—C3A	−161.8 (3)	O1C—Ni1C—O2C—C15C	−1.5 (2)
C1A—C2A—P2A—Ni1A	−34.4 (3)	P2C—Ni1C—O2C—C15C	176.5 (2)
C5A—C3A—P2A—C6A	−62.3 (4)	P1C—Ni1C—O2C—C15C	−6.9 (8)
C4A—C3A—P2A—C6A	173.7 (3)	C10C—C9C—P1C—C1C	−60.6 (3)
C5A—C3A—P2A—C2A	−173.0 (3)	C11C—C9C—P1C—C1C	175.8 (3)
C4A—C3A—P2A—C2A	63.0 (3)	C10C—C9C—P1C—C12C	−172.5 (3)
C5A—C3A—P2A—Ni1A	64.4 (4)	C11C—C9C—P1C—C12C	63.9 (3)
C4A—C3A—P2A—Ni1A	−59.5 (3)	C10C—C9C—P1C—Ni1C	61.4 (3)
O1A—Ni1A—P2A—C6A	52.5 (8)	C11C—C9C—P1C—Ni1C	−62.2 (3)
O2A—Ni1A—P2A—C6A	79.8 (2)	C2C—C1C—P1C—C9C	98.3 (3)
P1A—Ni1A—P2A—C6A	−97.68 (18)	C2C—C1C—P1C—C12C	−150.4 (3)
C15A—Ni1A—P2A—C6A	76.4 (2)	C2C—C1C—P1C—Ni1C	−28.2 (3)
O1A—Ni1A—P2A—C2A	165.2 (8)	C14C—C12C—P1C—C9C	65.5 (3)
O2A—Ni1A—P2A—C2A	−167.56 (19)	C13C—C12C—P1C—C9C	−167.6 (3)
P1A—Ni1A—P2A—C2A	14.98 (17)	C14C—C12C—P1C—C1C	−46.2 (3)
C15A—Ni1A—P2A—C2A	−170.9 (2)	C13C—C12C—P1C—C1C	80.7 (3)
O1A—Ni1A—P2A—C3A	−74.1 (8)	C14C—C12C—P1C—Ni1C	−166.1 (3)
O2A—Ni1A—P2A—C3A	−46.8 (2)	C13C—C12C—P1C—Ni1C	−39.2 (3)
P1A—Ni1A—P2A—C3A	135.75 (18)	O2C—Ni1C—P1C—C9C	67.8 (7)
C15A—Ni1A—P2A—C3A	−50.1 (2)	O1C—Ni1C—P1C—C9C	62.63 (18)
P1B—C1B—C2B—P2B	39.2 (3)	P2C—Ni1C—P1C—C9C	−115.47 (16)
O3B—C15B—Ni1B—O2B	102 (14)	C15C—Ni1C—P1C—C9C	62.0 (2)
O1B—C15B—Ni1B—O2B	−179.2 (4)	O2C—Ni1C—P1C—C1C	−172.2 (7)
O3B—C15B—Ni1B—O1B	−79 (14)	O1C—Ni1C—P1C—C1C	−177.35 (17)
O2B—C15B—Ni1B—O1B	179.2 (4)	P2C—Ni1C—P1C—C1C	4.55 (15)
O3B—C15B—Ni1B—P2B	101 (14)	C15C—Ni1C—P1C—C1C	−178.0 (2)
O2B—C15B—Ni1B—P2B	−0.6 (3)	O2C—Ni1C—P1C—C12C	−54.2 (7)
O1B—C15B—Ni1B—P2B	−179.80 (16)	O1C—Ni1C—P1C—C12C	−59.43 (17)
O3B—C15B—Ni1B—P1B	−79 (14)	P2C—Ni1C—P1C—C12C	122.47 (15)
O2B—C15B—Ni1B—P1B	178.89 (16)	C15C—Ni1C—P1C—C12C	−60.1 (2)
O1B—C15B—Ni1B—P1B	−0.4 (3)	C1C—C2C—P2C—C3C	−160.3 (3)
O3B—C15B—O1B—Ni1B	178.3 (4)	C1C—C2C—P2C—C6C	84.6 (3)
O2B—C15B—O1B—Ni1B	−0.7 (3)	C1C—C2C—P2C—Ni1C	−37.4 (3)
O2B—Ni1B—O1B—C15B	0.5 (2)	C4C—C3C—P2C—C2C	73.4 (3)
P2B—Ni1B—O1B—C15B	1.0 (8)	C5C—C3C—P2C—C2C	−164.4 (3)
P1B—Ni1B—O1B—C15B	179.7 (2)	C4C—C3C—P2C—C6C	−175.3 (3)
O3B—C15B—O2B—Ni1B	−178.3 (4)	C5C—C3C—P2C—C6C	−53.2 (3)
O1B—C15B—O2B—Ni1B	0.7 (3)	C4C—C3C—P2C—Ni1C	−46.3 (3)
O1B—Ni1B—O2B—C15B	−0.5 (2)	C5C—C3C—P2C—Ni1C	75.8 (3)
P2B—Ni1B—O2B—C15B	179.6 (2)	C8C—C6C—P2C—C2C	−159.7 (3)
P1B—Ni1B—O2B—C15B	−5.0 (7)	C7C—C6C—P2C—C2C	71.6 (3)
C11B—C9B—P1B—C12B	179.1 (3)	C8C—C6C—P2C—C3C	87.1 (3)
C10B—C9B—P1B—C12B	56.0 (4)	C7C—C6C—P2C—C3C	−41.7 (4)
C11B—C9B—P1B—C1B	−68.2 (3)	C8C—C6C—P2C—Ni1C	−41.5 (4)
C10B—C9B—P1B—C1B	168.7 (3)	C7C—C6C—P2C—Ni1C	−170.3 (3)
C11B—C9B—P1B—Ni1B	53.7 (3)	O2C—Ni1C—P2C—C2C	−164.43 (17)
C10B—C9B—P1B—Ni1B	−69.4 (4)	O1C—Ni1C—P2C—C2C	−153.7 (5)
C13B—C12B—P1B—C9B	−175.1 (3)	P1C—Ni1C—P2C—C2C	16.01 (15)
C14B—C12B—P1B—C9B	59.1 (4)	C15C—Ni1C—P2C—C2C	−161.6 (2)
C13B—C12B—P1B—C1B	71.8 (3)	O2C—Ni1C—P2C—C3C	−45.93 (17)
C14B—C12B—P1B—C1B	−54.0 (4)	O1C—Ni1C—P2C—C3C	−35.2 (6)
C13B—C12B—P1B—Ni1B	−48.8 (3)	P1C—Ni1C—P2C—C3C	134.52 (15)
C14B—C12B—P1B—Ni1B	−174.6 (3)	C15C—Ni1C—P2C—C3C	−43.1 (2)
C2B—C1B—P1B—C9B	101.0 (3)	O2C—Ni1C—P2C—C6C	80.43 (18)
C2B—C1B—P1B—C12B	−145.9 (3)	O1C—Ni1C—P2C—C6C	91.2 (5)
C2B—C1B—P1B—Ni1B	−23.5 (3)	P1C—Ni1C—P2C—C6C	−99.12 (16)
O2B—Ni1B—P1B—C9B	63.8 (6)	C15C—Ni1C—P2C—C6C	83.3 (2)
O1B—Ni1B—P1B—C9B	59.40 (18)		

### Hydrogen-bond geometry (Å, º)

**Table:**

*D*—H···*A*	*D*—H	H···*A*	*D*···*A*	*D*—H···*A*
C5*A*—H5*A*1···O3*A*^i^	0.98	2.7	3.670 (5)	169
C4*A*—H4*A*3···O1*A*^i^	0.98	2.69	3.448 (5)	134
C8*C*—H8*C*1···O3*C*^ii^	0.98	2.71	3.595 (5)	150
C10*B*—H10*F*···O3*B*^ii^	0.98	2.52	3.335 (5)	141
C1*A*—H1*A*2···O3*B*^ii^	0.99	2.23	3.204 (5)	168
C1*C*—H1*C*2···O3*A*^iii^	0.99	2.5	3.443 (5)	159
C9*C*—H9*C*···O2*A*^iii^	1	2.48	3.455 (5)	165
C1*B*—H1*B*1···O3*C*^iv^	0.99	2.5	3.452 (5)	161
C6*B*—H6*B*···O3*C*^iv^	1	2.6	3.516 (5)	153

Symmetry codes: (i) *x*+1, *y*, *z*; (ii) *x*−1, *y*, *z*; (iii) *x*+1/2, −*y*+1/2, *z*+1/2; (iv) −*x*+1, −*y*, −*z*+1.
